# Two novel mutations in *VPS33B* gene cause a milder ARC syndrome with prolonged survival in a 12-year-old patient: Case report

**DOI:** 10.3389/fped.2022.1041080

**Published:** 2022-12-07

**Authors:** Yingjie Zhu, Dongmei Chen

**Affiliations:** Department of Emergency/Critical Care Medicine, Children’s Hospital of Nanjing Medical University, Nanjing, China

**Keywords:** ARC syndrome, VPS33B, genetics, novel mutation, prolonged survival

## Abstract

Arthrogryposis–renal dysfunction–cholestasis (ARC) syndrome is a rare autosomal recessive disease caused by *VPS33B* and *VIPAR* gene mutations. The main clinical manifestations are congenital joint contracture, renal dysfunction mainly characterized by distal renal tubular dysfunction, and low glutamyltransferase cholestasis. Most patients with ARC die within 2 years of birth. Here, we report the case of a 12-year-old girl with an ARC phenotype who experienced long-term survival with only mild clinical symptoms. We detected two new heterozygous mutation sites of the *VPS33B* gene in this child, c.1081C > T (*p*.GLN361X, 257) and c.244T > C (*p*.Cys82Arg), through the gene detection technique; the tertiary structure of the protein was predicted by using the SWISS-model. We further reviewed the literature and summarized the clinical manifestations and gene loci of 19 ARC syndrome patients with long-term survival reported so far.

## Introduction

Arthrogryposis–renal dysfunction–cholestasis (ARC) syndrome (OMIM No. 208085) is a rare autosomal recessive inheritance disease, which was first reported in a child born from a consanguineous marriage. ARC syndrome is mainly concentrated in Arab countries where consanguineous marriages are common. Sporadic cases can also be seen in North Africa, European countries such as Spain, Italy, and Portugal, and Asian countries such as Japan and China. With the continuous popularization of genetic testing technology, the number of case reports of ARC syndrome has gradually increased, but its incidence is still unknown. ARC syndrome is a multisystem disease with three main characteristic symptoms, namely, congenital arthrogryposis, renal dysfunction dominated by distal tubular dysfunction, and low glutamyl transferase cholestasis. Other clinical features include dysplasia, ichthyosis and hyperkeratosis, hearing loss, platelet dysfunction, bleeding tendency, recurrent infection, and hypothyroidism. ARC syndrome is caused by mutations in vacuolar sorting–associated protein 33B (*VPS33B*) or *VIPAS39* (also known as VPS33B-interacting protein or apical–basolateral polarity regulator) (1). *VPS33B* gene is located on chromosome 15q26.1, with a length of 23.9 kb and 23 exons. The encoded *VPS33B* protein is mainly involved in intracellular protein transport. *VPS33B* gene is expressed in the fetal and adult bone, kidneys, liver, and skin. Therefore, ARC syndrome patients have impaired structure and function of motor units, renal tubules, hepatic lobules, and epithelial cells. As a result, joint contracture, amino aciduria, glycosuria, cholestasis, ichthyosis, and other multisystem manifestations can occur. Due to recurrent infection, acidosis, or severe bleeding, most ARC syndrome patients do not reach their second year of life (2, 3). However, recently, reports of children with long-term survival have been increasing (4). Here, we report a patient who was admitted to our hospital with a complaint of yellow skin staining along with icteric sclera, contracture of finger and toe joints, dry skin, and signs of ichthyosis. Further examination revealed hyperbilirubinemia and low GGT cholestasis, which could not be reduced during the patient's 10-day stay at the hospital. Whole exon gene sequencing revealed that the patient had late-onset ARC. Unlike typical cases, the patient showed no indications of ARC at birth or infancy. We inferred that, unlike typical cases, the patient had mild symptoms and prolonged survival due to two new *VPS33B* gene mutations.

## Case description

The patient was a 12.9-year-old girl from Mainland China, who presented with yellow skin staining that she had for 5 days. she was the only child to the parents, who were not consanguineous. Furthermore, the patient's mother had a full-term normal pregnancy and was not diagnosed with any abnormalities. The patient's birth weight was 3.5 kg. Apart from mild joint contracture, no abnormalities were detected after birth, and she experienced normal growth and development. Her first hospital visit was at the age of 5 when she was diagnosed with ichthyosis because of repeated dry and itchy skin with desquamation. There was no significant improvement in this condition even after she was administered external and oral drugs.

**Figure 1:**
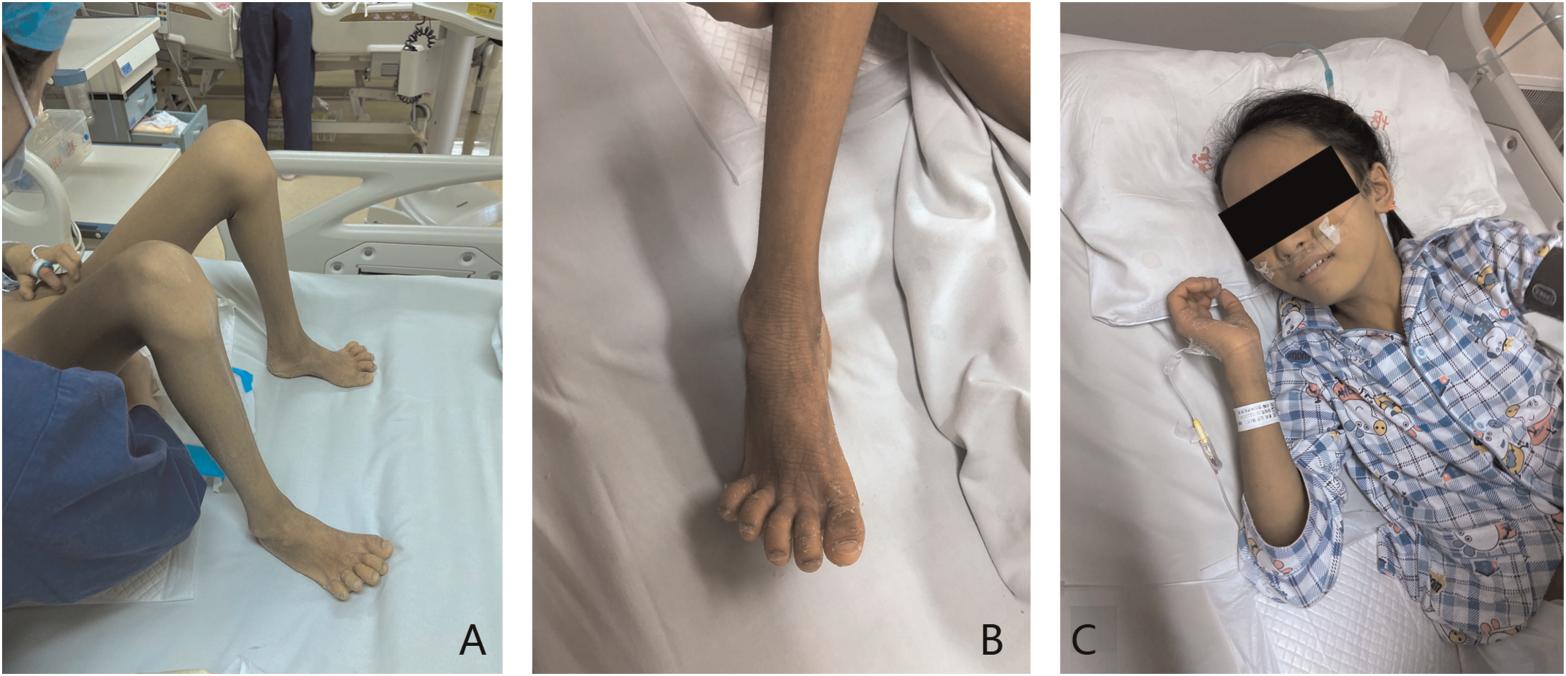
Clinical symptoms. (**A**) Joint contracture can be seen in the finger and toe joints of the patient. (**B**) The patient has dry skin and hand and foot desquamation, which is the manifestation of ichthyosis. (**C**) The patient has a yellow skin stain all over the body, accompanied by icteric sclera.

In this second hospital visit, the patient was brought with a complaint of yellow skin staining, accompanied by obvious hunger. Physical examination showed yellowing all over the body, accompanied by icteric sclera, contracture of the finger and toe joints, dry skin all over the body accompanied by incessant itching, and hand and foot desquamation, which is a manifestation of ichthyosis, as shown in Figure 1.

The outpatient examination showed 230.2 μmol/L TBil, 26.7 μmol/L IBil, 32.9 g/L albumin, 31 U/L AST, 32 U/L ALT, 16 U/L GGT, and 173 μmol/L bile acid. Her renal and coagulation functions were normal; routine urine tests showed high urine bilirubin, moderate levels of urine protein, and normal urine sugar. Cranial MRI showed a slightly higher signal in the bilateral basal ganglia on T1WI. Abdominal B-ultrasound showed mild liver enlargement and abdominal CT showed full shadows in the liver and pancreas and small flaky high-density shadows in the bilateral kidneys. MRCP was normal, ruling out any organic disease of the biliary tract.

The patient was admitted after these tests. In addition to all the other symptoms, at the time of admission, we observed that the child was irritable and had a speech disorder. We speculated that this might be related to bilirubin encephalopathy. As part of the treatment process post-admission, the patient was given ursodeoxycholic acid, ornithine aspartate injection, lactulose, probiotics, atomolan, compound glycyrrhizic anhydride, fresh frozen plasma, and a non-protein diet, but there was no significant improvement in her hyperbilirubinemia and low GGT cholestasis. This finally led us to conduct a whole exon gene sequencing to shed light on this unexplained juvenile cholestasis. While awaiting the sequencing results, we started eight plasma transfusions on the 3rd day of her admission, but the bilirubin and bile acid of the patient decreased only slightly and increased again after the plasma exchange stopped (Table 1).

**Table 1:** Changes of transaminase, bile acid, and bilirubin in the patient.

Hospital days	ALT (U/L) [7–30]	AST (U/L) [14–44]	TBiL (μmol/L) [3.4–17.1]	DBiL (μmol/L) [0–6.8]	IBiL (μmol/L) [1.7–13.7]	GGT (U/L) [5–19]	TP (g/L) [61–79]	Bile acid (μmol/L) [<10]
0	31	32	230.2	126.7	103.5	16	32.9	–
2	19	23	355.73	285.19	70.54	16	28.1	173.0
4	10	17	377.64	297.61	80.03	20	35.0	–
6	7	13	339.82	273.81	66.01	19	32.7	–
7	8	16	373.77	296.2	77.57	19	33.9	151.1
9	4	9	275.98	223.42	52.56	14	29.9	–
11	10	12	193.88	160.79	33.09	15	33	170.1
13	15	15	189.48	156.4	33.08	17	34.8	–
15	18	16	238.82	193.5	48.06	21	38.2	188.9
17	18	18	258.82	210.76	45.32	24	40.2	–
19	14	14	240.99	199.89	41.1	20	36.2	239.9
21	20	16	255.57	211.96	43.61	21	35.7	–
22	25	21	265.97	218.53	47.44	20	36.4	264.6

ALT, alanine transaminase; AST, aspartate transaminase; TBiL, total bilirubin; DBiL, direct bilirubin; IBiL, indirect bilirubin; GGT, G-glutamyltransferase; TP, total protein.

On the 10th day after admission, the child developed a fever and was given anti-infection treatment. Because plasma exchange could not reduce the bilirubin level, we suggested liver transplantation. However, her parents rejected this suggestion and chose to leave our hospital; the patient then underwent a liver puncture examination at another hospital; the pathological results showed intrahepatic bile duct stenosis. We later followed up with the patient and learned that her bilirubin and bile acid levels had gradually decreased upon treatment with oral ursodeoxycholic acid; our most recent follow-up revealed that the patient's bilirubin and bile acid levels were normal, but the skin symptoms did not improve.

**Figure 2:**
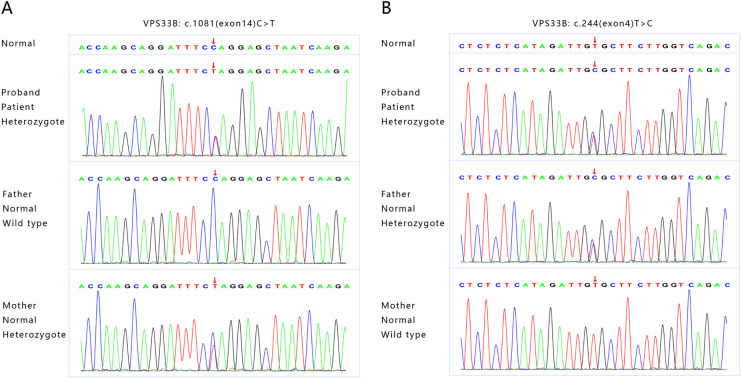
The whole exon gene sequencing showing two novel heterozygous mutations in *VPSS33B* gene. (**A**) The first mutation is c1081(exon14)C > T, NM_018668, *p*.Q361X,257, which originated from the patient's mother. (**B**) The second mutation is c.244(exon4)T > C, NM_018668, *p*.C82R, which originated from the patient's father.

Finally, the whole exon gene sequencing of the patient revealed that she had two heterozygous mutations in the *VPSS33B* gene. The first was c.1081(exon14) C > T, NM_018668, *p*.Q361X,257. Following ACMG guidelines, the mutation was determined to be pathogenic (PVS1 + PM2 + PP3), which was inherited from her mother. The second was c.244(exon4) T > C, NM_018668, *p*.C82R, which, based on ACMG guidelines, was determined to be a likely pathogenic mutation (PVS1 + PM2 + PM3 + PP3), inherited from her father (Figure 2). These two newly discovered gene mutations may be related to the mild ARC syndrome reported previously.

## Discussion and conclusions

**Figure 3:**
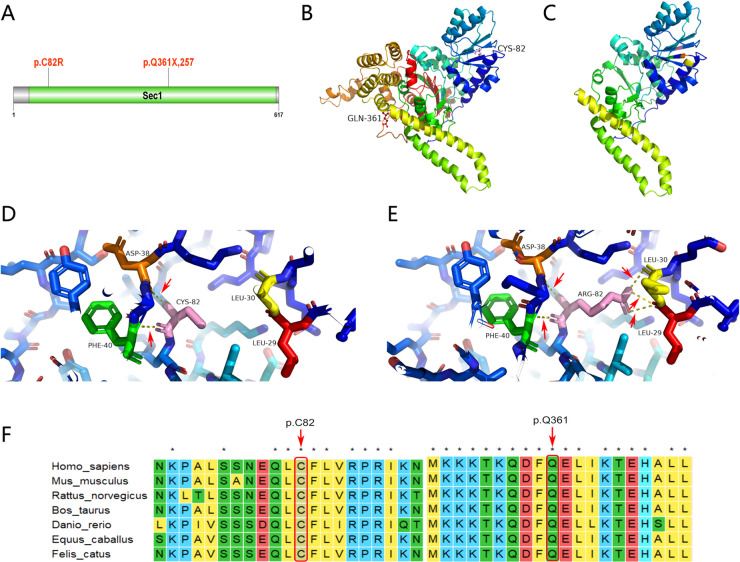
Genetic analysis and illustration of the heterozygous c1081C > T and c.244T > C mutation of *VPSS33B* gene. (**A**) VPS33B protein secondary structure; *p*.Q361X, 257 and *p*.C82R are located in the Sec1 domain. (**B**) VPS33B protein 3D structure (wild-type global image): Gln361(red) and Cys82 (pink). (**C**) VPS33B protein 3D structure (mutant global image); termination of amino acid translation at position 361 of VPS33B protein, resulting in the loss of 257 amino acids and truncated protein. (**D**) VPS33B protein 3D structure (wild-type local image); Cys82 (pink) forms a hydrogen bond with Leu29 (red) and Leu30 (yellow); the red arrow points to the hydrogen bond (yellow dotted line). (**E**) VPS33B protein 3D structure (mutant local image); Arg82 (pink) forms a hydrogen bond with Leu29 (red), Leu30 (yellow), Asp38 (orange), and Phe40 (green), respectively; the red arrow points to the hydrogen bond (yellow dotted line). Compared with the wild type, the number of hydrogen bonds has increased and the protein conformation has changed. (**F**) Conservation analysis of VPS33B protein; Gln361 and Cys82 are located in the highly conserved amino acid region, and the conserved amino acid mutation may damage the protein function.

Unlike typical ARC that occurs immediately after birth, clinical manifestations in our patient occurred at different stages of life. Joint contracture occurred after birth, ichthyosis occurred at the age of 5, and cholestasis, elevated bilirubin, and mild proteinuria occurred at the age of 12. Such differences in manifestation may be linked to new, previously unknown gene mutations. At present, 75% of patients with ARC syndrome have the *VPS33B* gene mutation. The gene is located at chromosome 15q26.1 and encodes a VPS33B protein consisting of 617 amino acids (1). We found two related gene mutations by the whole exon gene sequencing of this patient, which have not been reported so far. The first is c.1081C > T (*p*.Q361X,257), a heterozygous mutation located in the 14th exon of the *VPS33B* gene, inherited from her mother. This mutation indicates that cytosine at position 1,081 of the *VPS33B* gene coding region is replaced by thymine, resulting in the termination of amino acid translation at position 361 of VPS33B protein, the loss of 257 amino acids, and the protein being truncated (Figure 3B shows the wild-type *VPS33B* gene; Figure 3C shows the mutation). The second, c.244T > C (*p*.C82R), is also a heterozygous mutation, located in the fourth exon of the *VPS33B* gene, originating from the patient's father; this indicates that the thymine at position 244 of the *VPS33B* gene coding region has been replaced by cytosine, resulting in cysteine residue at position 82 of the tertiary structure of the VPS33B protein replaced by arginine (*p*.C82R). We used a SWISS-Model (swissmodel.expasy.org) to predict the tertiary structure of the protein. The results showed that c.1081C > T caused the early termination of amino acid coding, resulting in significant changes in the protein structure. c.244T > C makes Arg82, which replaces Cys82, forming a hydrogen bond with Leu29, Leu30, Asp38, and Phe40, respectively, while Cys82 forms a hydrogen bond only with Leu29 and Leu30 in the wild type. Compared with the wild type, the number of hydrogen bonds increased and the protein conformation changed, as shown in Figures 3D,E. Using software, we also identified that Gln361 and Cys82 were located in the highly conserved amino acid region, and the conserved amino acid mutation was likely to damage the protein function, as shown in Figure 3F.

VPS33B is homologous with yeast Sec-1/Munc18 family protein VPS33P. The secondary structure analysis of the VPS33B protein in this patient shows that *p*.Q361X,257 and *p*.C82R are indeed located in the Sec1 protein domain (Figure 3A). Sec-1/Munc18 protein (SM protein) regulates vesicle trafficking and fusion by binding to the T-SNARE of the Syntaxin family in a variety of modes, and SM protein plays an important role in membrane protein trafficking and maintenance of cell polarity (5, 6). This protein binds and modulates the conformation of SNARE protein, which is homologous to neuronal synaptic membrane protein syntaxin and is critical for neurotransmitter release and other secretory events (7). It is possible that our patient's irritability is related to mutations in this region. Current studies have found that both *VPS33B* and *VIPAR* interact with the HOPS complex protein VPS18. *VIPAR* may interact with VPS18 only in the absence of *VPS33B*, so the *VPS33B-VIPAR* complex is unlikely to participate in the HOPS interaction (8). However, *VIPAR* may interact with VPS18 in mutant *VPS33B* cells, implying that this interaction may be involved in the pathogenesis of ARC syndrome. The *VPS33B* gene is expressed in the bone, kidneys, liver, and skin; thus, the clinical manifestations show multisystem involvement (9).

Hanley et al. used a VPS33Bfl/fl-AlfpCre mouse model and found that VPS33B is crucial for maintaining the structure and function of mammalian hepatocytes. Patients with ARC syndrome with *VPS33B* mutation have cholestasis, high plasma ALP activity, and high bile acid level, while transaminase activity and GGT levels remain normal. This is because of an incorrect localization of apical membrane proteins, such as ARE, in hepatocytes but a normal localization of basolateral membrane proteins in hepatocytes (10). The study also found that VPS33B affects bile acid homeostasis and lipid metabolism in mice and contributes to the progress of liver cholestasis. VPS33B affects the progress of liver metabolism in both bile acid circulation and lipid metabolism, while VPS33B deficiency plays a key role in aggravating cholestasis and liver injury (7, 11).

We summarized the histories of patients with mostly mild and partial clinical manifestations of ARC syndrome, who were reported to have achieved long-term survival thus far, as shown in Table 2. Surprisingly, in the acute phase, we could not alleviate low GGT cholestasis and hyperbilirubinemia through plasma exchange in our patient, but the child's bilirubin and bile acid indices returned to normal with just oral drug treatment after discharge, suggesting that hyperbilirubinemia and cholestasis may have been paroxysmal. Similar histories were reported in other cases. For instance, patient number 7, whose history is summarized in Table 2, presented with a spontaneous resolution of hyperbilirubinemia. To understand why such a phenotype exists, it may be necessary to further study the upstream and downstream regulation of relevant genes.

**Table 2:** Clinical manifestations of ARC syndrome in patients with prolonged survival.

Patient	Consanguineous mating	Ethnic	Gender	Arthrogryposis	Renal dysfunction	Cholestasis	Include ichthyosis/hyperkeratosis	Platelet dysfunction	Sensorineural deafness	Agenesis of the corpus callosum	Severe failure to thrive	Recurrent infection	survival
1 (12)	+	Spanish	M	+	–	+	–	–	+	–	+	–	alive at 3y
2 (4)	+	Arab	F	+	+	+	–	N	–	+	+	–	alive at 7.7y
3 (4)	+	Arab	M	+	+	+	–	N	–	+	+	–	alive at 3.3y
4 (13)	–	Mexico	F	–	+	–	–	+	–	–	+	+	died at 8.5y
5 (14)	–	Spanish	F	+	+	+	+	+	–	+	+	+	alive at 6y
6 (15)	+	Brazil	M	+	–	+	+	–	–	+	+	–	alive at 17y
7 (15)	+	Brazil	F	–	–	+	+	–	–	–	+	–	alive at 10y
8 (15)	–	Brazil	M	–	–	+	+	+	+	–	+	–	alive at 11y
9 (16)	–	Japan	M	–	Proteinuria	–	–	–	+	–	+	–	alive at 6y
10 (16)	–	Japan	M	–	+	+	–	–	+	–	–	–	alive at 24y
11 (17)	–	Spanish	F	+	+	–	+	–	+	–	–	–	alive at 12y
12 (18)	+	Austria	M	–	Proteinuria	–	+	–	+	–	–	–	alive at 13y
13 (18)	–	Austria	F	+	–	–	+	–	+	–	+	–	alive at 35y
14 (18)	–	Austria	M	+	–	–	+	–	+	–	+	–	alive at 59y
15 (8)	–	Peru/Puerto Rico	M	+	+	+	–	+	+	–	+	–	alive at 5.5y
16 (8)	N	Puerto Rico/Mexico	F	+	+	+	+	–	+	+	+	–	alive at 3.5y
17 (19)	+	China	M	–	Proteinuria	+	–	–	–	–	–	–	alive at 7y
18 (19)	–	China	M	–	Proteinuria	+	–	–	–	–	–	–	alive at 4y
19 (19)	–	China	F	–	Proteinuria	+	–	–	–	–	–	–	alive at 9y

At present, among the gene mutations that are related to prolonged survival of patients with ARC, five were found to have c.1225 + 5G > C gene mutations (Table 3). Previously, it was also reported that c.1225 + 5G > C mutations are related to mild ARC syndrome. *VPS33B* c.1225 + 5G > C mutation produces a truncated VPS33B protein (residue 1,420) that is still partially co-domain with VIPAR. At the same time, cell experiments show that the c.1225 + 5G > C mutant still retains a part of the ability to interact with VIPAR, but the mutant affects the ability of the complex to co-locate on tubular vesicular circulating membrane, which at least partially impairs their cell function (8). Two gene mutation sites in our patient, c.1081C > T and c.244T, caused the deletion of C-terminal amino acids of the protein, but the N-terminal coding was not affected. c.244T > C also increased the number of hydrogen bonds in the tertiary structure of the protein and did not change the overall structure of the protein, which may have helped preserve the function of VPS33B protein, resulting in a mild phenotype of ARC and prolonged survival in the patient.

**Table 3:** Currently known *VPS33B* gene mutation sites associated with prolonged survival in ARC syndrome.

Patient	Gene	Mutation sites	Hom/Het	Amino acid
1 (12)	*VPS33B*	c.1225 + 5G > C		
		c.1246C > T	hom	p.R416X
2 (4)	*VPS33B*	c.1157A > C	hom	p.His386Pro
3 (4)				
4 (13)	*VPS33B*	c.1609_1657 + 9del		
		c.1225 + 5G > C		
5 (14)	*VPS33B*	c.1225 + 5G > C	het	
		c.440_499del	het	Pro147Argfs*4
6 (15)	*VPS33B*	c.1148T > A	hom	p.(Ile383Asn)
7 (15)	*VPS33B*	c.1148T > A	hom	p.(Ile383Asn)
8 (15)	*VPS33B*	c.1148T > A	het	p.(Ile383Asn)
		c.940-2A > G	het	
9 (16)	*VPS33B*	c.403 + 2T > A	het	
		c.1582-9C > G	het	
10 (16)	*VIPAS39*	c.339del	het	p.(Phe113Leufs*60)]
		c.1035C > G	het	p.(Tyr345*)
11 (17)	*VPS33B*	c1225 + 5G > C		
12 (18)	*VPS33B*	c.[390G > A;392G > A]	hom	p.Gly131Glu
13 (18)	*VPS33B*	c.[390G > A;392G > A]	hom	p.Gly131Glu
14 (18)	*VPS33B*	c.[390G > A;392G > A]	het	p.Gly131Glu
		c.240-1G > C	het	
15 (8)	*VPS33B*	c.240–577_290-156del	het	p.Gln421Valfs∗8
		c.1225 + 5G > C.		
16 (8)	*VPS33B*	c.1261_1262delCA	het	p.Gln421Valfs∗8
		c.1225 + 5G > C		
17 (19)	*VPS33B*	c.1726T > C	hom	p.Cys576Arg
18 (19)	*VPS33B*	c.1726T > C	het	p.Cys576Arg
19 (19)	*VPS33B*	c.1726T > C	het	p.Cys576Arg

Our discovery of two new gene mutation sites in an ARC patient with prolonged survival further suggests that other unexplained cholestasis in older children may be linked to ARC gene mutation. Furthermore, missense mutations were common in other ARC syndrome patients who had prolonged survival (Table 3).

At present, there is no clear and effective treatment for ARC syndrome. Most such patients are given supportive treatments such as fluid supplementation, anti-infection, and enteral nutrition to relieve acidosis, hyperbilirubinemia, and recurrent infection in order to stabilize their condition and improve their quality of life. Although there is no effective gene therapy for ARC syndrome at present, it is believed that with the deepening of molecular genetics research and the development of clinical diagnosis and treatment technology, gene therapy will bring new hope to the prevention and treatment of ARC syndrome. We hope our findings can support developments in gene therapy for patients with mild ARC.

## Data Availability

The datasets presented in this study can be found in online repositories. The names of the repository/repositories and accession number(s) can be found in the article/Supplementary Material.
